# Effect of Perceptual Training with Sound-Guided and Kinesthetic Feedback on Human 3D Sound Localization Capabilities

**DOI:** 10.3390/s23115023

**Published:** 2023-05-24

**Authors:** Ranjita Kumari, Sukhan Lee, Jonghwan Shin, Soojin Lee

**Affiliations:** 1 Department of Electrical and Computer Engineering, Sungkyunkwan University, Suwon 16419, Republic of Korea; ranjita@skku.edu (R.K.); hwanyy@skku.edu (J.S.); 2 Department of Artificial Intelligence, Sungkyunkwan University, Suwon 16419, Republic of Korea; christie74@skku.edu

**Keywords:** blind person, vision system, sound localization, perceptual training, kinesthetic assistance

## Abstract

In this paper, we experimentally investigate how the 3D sound localization capabilities of the blind can improve through perceptual training. To this end, we develop a novel perceptual training method with sound-guided feedback and kinesthetic assistance to evaluate its effectiveness compared to conventional training methods. In perceptual training, we exclude visual perception by blindfolding the subjects to apply the proposed method to the visually impaired. Subjects used a specially designed pointing stick to generate a sound at the tip, indicating localization error and tip position. The proposed perceptual training aims to evaluate the training effect on 3D sound localization, including variations in azimuth, elevation, and distance. The six days of training based on six subjects resulted in the following outcomes: (1) In general, accuracy in full 3D sound localization can be improved based on training. (2) Training based on relative error feedback is more effective than absolute error feedback. (3) Subjects tend to underestimate distance when the sound source is near, less than 1000 mm, or larger than 15° to the left, and overestimate the elevation when the sound source is near or in the center, and within ±15° in azimuth estimations.

## 1. Introduction

As per the international agency for the prevention of blindness (IAPB), around 43 million people were completely blind in 2020, and approximately 61 million will be blind in 2050 [1]. The most serious problem encountered by the blind is mobility. Blind people are out of touch with the surrounding environment. They do not see the things happening around them and need time to realize what happened. While walking on the roads, they face many problems, such as obstacles, collisions with others, encountering manholes, etc. All blind people seek an excellent visual support system using the current abundance of technologies to fulfill their basic needs in daily life.

Visually impaired people have used sticks to guide their way for a long time. It has several limitations, such as detecting obstacles and manholes in time. A smart blind stick using assistive technologies such as ultrasonic sensors or cameras to detect obstacle heights and a laser sensor for detecting manholes has improved the mobility of visually impaired people [2]. However, this may only work in some scenarios and situations. Visually impaired people need assistive technology beyond the smart blind stick, which can provide current location information to the surrounding environment. Mobile computerized devices can generate navigation instructions for the surrounding environment providing visually impaired people the chance to navigate their surrounding environment [3,4,5]. A 3D smartphone and various algorithms (such as stereo vision and dense disparity map) can detect obstacles and provide essential information for visually impaired people [6,7]. Once an obstacle is detected, acoustic feedback such as beep sounds with different frequencies and repetitions is provided to blind people to prevent a collision.

Research has progressed, and researchers worldwide have realized that providing sound-guided information alone is not enough for the blind person’s navigation. Hence, the increase in the research in the direction of mapping 3D space in vibration and audio format using various sensing and image processing technologies, and the power of artificial intelligence to give a sense of environmental space [8,9,10,11]. However, before developing a vision system, we need to know about the strength and weaknesses of visually impaired people. The strength of the blind is that they are sensitive to sound and touch [12,13]. Several kinds of research have demonstrated that touch provides a more profound knowledge of reality than audio for the visually impaired [14,15,16].

Meanwhile, functional magnetic resonance imaging (fMRI) techniques have evolved to study the human brain. To develop a realistic vision support system, researchers have proposed studies to understand the cross-modal plasticity [17,18,19,20] and neural plasticity of the blind brain [21,22]. Several studies have confirmed that blind people have superior abilities in non-visual perceptual tasks, verbal memory [23], and sound localization [24]. In addition, it was confirmed that the visual cortex is activated during an auditory task in the blind [25]. Based on these research findings, researchers are using the superior ability of the blind to develop a non-visual device that provides them with a pseudo-visual sensation [26,27]. However, blind people need systematic training to adapt to these pseudo-vision systems while enhancing their skills to use them effectively. Systematic training to improve the pseudo-3D vision abilities of the blind [28,29,30,31], here referred to as perceptual training, should be designed based on scientific, cognitive, and behavioral psychology.

Perceptual training is the process that improves performance through sensory interaction with the environment as well as through practice with specific sensory tasks [32,33]. Perceptual training improves people’s abilities through experience. Each repetition or recall of the knowledge acquired through perceptual training strengthens the pattern and makes it more efficient. Any patterns become more robust with more repetition and are weakened by no use.

In this paper, we are interested in developing systematic perceptual training for the pseudo-vision ability of the blind; in particular, by combining perceptual training with kinesthetic assistance. Through experimentation and learning, kinesthetic assistance improves critical thinking and analytical skills [34]. It is an active type of learning that uses body movements such as hand movements. The more engaged an organism is, the better the brain learns when exploring. The cerebellum is more active during kinesthetic assistance as it engages in motor skills. Hence, multi-modal perceptual training with kinesthetics is very effective for training new skills such as sound localization and adopting a pseudo-vision system for blind people.

The rest of the paper is organized as follows. Section 2 provides related work. Section 3 describes the method and materials. It describes the subjects, test setup, test procedure, data collection, statistical analysis, and pretest result approach. The detailed test results are described in Section 4. This section further divides into three subsections to analyze the result in terms of distance, elevation, and azimuth error. Section 5 has a detailed discussion about the training method and its efficacy based on the result. Finally, Section 6 concludes the proposed training method by discussing future work.

## 2. Related Works

Sound localization was studied in six sighted people in a natural environment with paired auditory and visual feedback [35]. This study was performed in eight different spatial regions (four in front and four behind the subjects). Each region had a sound source at nine different locations. The test was conducted in two sessions. The sound source location was fixed at a distance of 150 cm. The overall localization error was reduced from 40° to 25° after the training. This result shows that feedback training improves localization accuracy in sighted subjects. Moreover, as per the authors, the subject’s ability to estimate the spatial location of the sound source does not have a short-term effect but stays several days after the training. Since this sound localization training was performed with sighted subjects, we further explored more research papers.

A comparative study was performed between four blind and four blindfolded (sighted) people to understand their sound localization acuity [36]. The study was performed under two conditions in a real environment: head fixed and head rotation. In an experimental room, 12 loudspeakers were arranged in a circle at a fixed 150 cm distance. The training was performed in five sessions. Each session had 12 trials. The study shows that the mean localization error for the blind was 4.2° and 3.5° compared to blindfolded at 8.8° and 7.8° for the head fixed and head rotated conditions, respectively. The average distance error for the blind was +18 cm and +71 cm compared to those blindfolded of −81 cm and −84 cm, respectively, for the head fixed and rotated head. This study shows that the acuity of the blind is significantly higher than sighted people in both azimuth and distance estimation. This sound localization training was performed without any feedback to the participants.

Some more sound localization studies were performed based on multi-model perceptual feedback consisting of auditory, haptic, and visual feedback. This study integrated 3D binaural sounds with 21 sighted subjects in a virtual environment from non-individualized head-related transfer functions (HRTF). In this experiment, subjects indicated the sound location from 0° and 355° on a computer in a circle using a mouse cursor based on ten different heard sounds from ten different places. The study showed that the average azimuth error reduced from 55.38° to 45.85°. This research continued, and a study was performed with nine sighted and nine visually impaired people [31]. Different sound stimuli were played from 12 different locations from 0° to 360° with 30° increments. The training was conducted in two blocks of 12 rounds in each block. In each round, blind subjects had to indicate the sound source using the conventional hour hand mark of the clock, and sighted subjects had to indicate the sound source on the computer using a mouse cursor in a circle. This research found that sound localization training with feedback improved spatial auditory resolution and orientation in sighted and blind subjects.

### 2.1. Problem Statement

The human ability for 3D sound localization has been studied comprehensively in sighted, blindfolded, and blind subjects. Sound perception in 3D space by humans is more complex than thought. The time lag (also known as the interaural time difference) for sound to reach the left and right ears gives the azimuth perception. In contrast, elevation perception links to spectral differences associated with the geometric structure of the ears. The volume or intensity difference of a sound gives the distance perception. However, not much study has been conducted on how 3D sound localization training affects the human capacity to recognize sound accurately regarding distance, elevation, and azimuth. Moreover, which training method is more effective in improving sound localization, and how sound localization training takes advantage of neuroplasticity in our brain also need to be well-researched. In particular, for any training, feedback plays an essential role in its effectiveness. However, conventional research needs to give proper attention to the effect of feedback types on training effectiveness.

### 2.2. Contribution

We propose a new approach for sound localization training based on perceptual training with sound-guided feedback and kinesthetic assistance to proceed further beyond improving the subject’s ability to locate the sound source accurately. In our proposed training, the subject is guided through interactive sound feedback and vibration using kinesthetics to locate the sound source accurately. To be precise our contributions are summarized below.

- We exclusively investigated the effect of training on improving human ability in 3D sound localization. In particular, unlike conventional works, our proposed training considered full 3D sounding in training, not only azimuth and elevation but also distance.
- In recognition of the importance of feedback in training, we devised several feedback mechanisms to investigate their effect on training efficacy. In particular, we focused on sound and kinesthetic feedback, instead of visual feedback, to accommodate the visually impaired. Especially, in devising feedback mechanisms, we consider externally provided guidance to reach the target with the relative error feedback, besides the absolute error feedback, to investigate for the first time the effect of relative error feedback in the training of 3D sound localization.
- We successfully implemented the proposed training on blindfolded subjects and improved their sound localization ability.

## 3. Materials and Methods

Perceptual training with kinesthetic assistance-based sound localization training starts with considering different aspects such as subjects involved in this study, test preparation, test setup, test environment, test procedure, data collection, and statistical method involved to analyze the data. Each of these is elaborated on in subsequent sections.

### 3.1. Subjects

Six adults (20–40 years) were recruited as participants. All participants had normal hearing. Subjects included males (*n* = 4, 20–40 years) and females (*n* = 2, 20–35 years). We obtained written informed consent from each to participate actively in this research.

### 3.2. Test Setup

**Figure 1:**
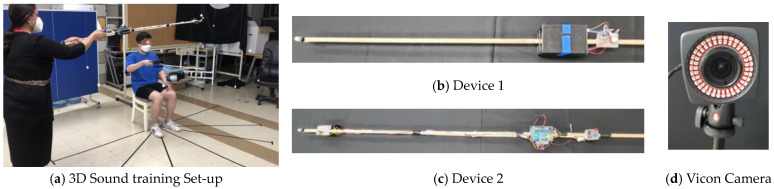
Three-dimensional sound localization experimental training setup. (**a**) Three-dimensional sound training setup to demonstrate training methodology. The experimenter has device 2 in hand placing the sound source in 3D space. Subject with device 1 pointing to the sound source location. (**b**) Device 1 with ATmega328P microcontroller with vibrator and push button. (**c**) Device 2 with ATmega328P microcontroller and 3 W stereo enclosed speaker. (**d**) Vicon camera (four quantities). All these training procedures were recorded by a personal computer with a Nexus 1.8.3 program.

The subjects were blindfolded outside the experimental room (in another room) and exposed to the experimental environment. The experimental room consisted of predefined land markings in space that used small objects hanging by a string from the ceiling at various heights and distances, in such a way as to provide the experimenter with the perception of 3D experimental space and guidance for uniform distribution of sound sources during the experiment. The blindfolded subject was seated in the center of the experimental room, and sound stimuli were played from different locations. The entire space was divided into different segments for better analysis of the results. Based on distance, we divided the space into near (for less than equal to 1 m) and far (for greater than 1 m). Considering azimuth, we divided the space into the left (less than 15°), center (in between ±15°), and right (greater than 15°) areas. Considering the elevation, the interval was divided into up (above the subject’s shoulder) and down (below the subject’s shoulder). Our research confirmed that sound stimuli should be tested at different locations in these areas during testing. The test was conducted in uncontrolled environmental conditions with natural sound during the experiment. We replicated a natural and practical testing environment to ensure the applicability of the training in real life. As displayed in Figure 1, our proposed training setup consists of an experimenter with device 2, a subject with device 1, a test environment with four Vicon cameras, and a personal computer with a Nexus 1.8.3 program. Device 1 consists of an ATmega328P microcontroller with a vibrator to provide kinesthetic movements, a speaker for sound guidance feedback, and a push button to respond to an experimental beep sound. Device 2 is designed with an ATmega328P microcontroller and a 3 W stereo speaker on another stick. The speaker on device 2 produces the target sound.

### 3.3. Test Procedure

On each trial, experimenter one brings each blindfolded subject (one at a time) inside the experimental room and makes him/her sit in the predefined location. We ensure that the subject feels comfortable prior to the experiment. Experimenter 2 instructs the subject about the purpose of the experiment and the experimental procedure. Experimenter 2 teaches the subject how to use device 1 to estimate the sound source location he/she hears. Subjects are briefed about different sounds, which are played during the experiment from device 2. A target sound is a source stimulus that the subject has to localize at a particular location. Using device 2, a target sound of 80 dB amplitude and 500 Hz frequency is played for 500 ms duration five times in 1.5 s intervals. We tested various amplitudes and frequencies of sound for their suitability as a sound source for the experiment by using many subjects prior to the start of the experiment. We received feedback from participating subjects that a test stimulus with 80 dB and 500 Hz is the most comfortable sound for various positions in 3D space. Therefore, we used a sound stimulus of 80 dB amplitude and 500 Hz frequency. We generated this sound by Piezo. This is a vibration-based sound source such as a flute, not too low, not too high. It is a mild sound.

We stopped playing the target sound, and then the subject responded to location of the sound source by pointing in the direction using device 1. Suppose the subject can locate the sound source within a 150 mm distance error. In that case, a known success sound is generated for 2 s to give the subject the information that he/she succeeded in the localization test. Hence, the test terminates for this particular test stimuli. Otherwise, a known failure sound for 2 s is produced to give feedback to the subject that he/she failed in the localization test for this particular test stimuli. At this stage, we play a guidance sound of 80 dB amplitude and varying frequency. Guidance sound frequency changes in real-time based on the relative distance error in millimeters (mm) multiplied by 1.5. The more distance error, the higher the guidance sound frequency. Therefore, the device 1 speaker produces a different pitch for the guidance sound for the subject to understand how far their stick pointer is from the target (sound stimuli played before). This helps the subject to navigate using device 1 to reach the target.

In each trial, we conducted the training by keeping sound stimuli at different locations. The training period spanned three weeks, consisting of two sessions per week held on Tuesdays and Fridays. Each training day session had 5 trials and each trial had 6 test stimuli from different locations for every subject. Therefore, there existed a 3- or 4-day gap between the two consecutive trials, from which we could glimpse a short-term forgetting effect. The average performance of the 1st trials, representing the performance after short-term forgetting, tends decrease over six sessions, indicating some retainment of the training effect over time. Repeated testing and corrective feedback reinforce the subject to enhance sound localization capabilities.

**Figure 2:**
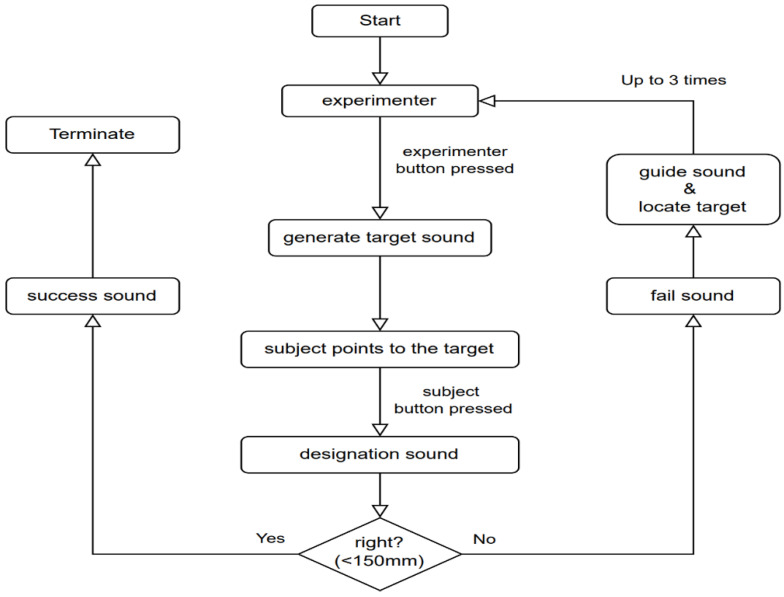
Experimental steps of 3D sound localization training.

As illustrated in Figure 2, sound localization training steps are as follows.

- Once the subject is ready, experimenter 1 presses the button to play the target sound from device 2.
- The subject listens to the target sound and tries to locate the target sound by device 1.
- The subject presses the button on device 1 to show their response to locate the target sound. Once the subject presses the button, a designation sound is generated. A designation sound can be a success sound or a failure sound.
- Our internal goal is to reduce the mean distance error below 250 mm derived from the under-developed vision system, so we decided to keep our training goal as a distance error of less than 150 mm. If the subject locates the target sound source within 150 mm proximity, then a successful sound is generated right away. In this case, the experiment proceeds to the next test location.
- The speaker generates a guidance sound if the subject fails to locate the sound source. A high pitch sound is generated if device 1 is far from the target sound. A low-pitch sound is generated if device 1 is near the target sound.
- Once the subject locates the target sound source based on guidance sound, a booming sound is generated.
- The subject must fold his/her arms to the rest position after finishing each test stimuli experiment.
- The total number of trials is five, each with six different test stimuli at different locations. Thus, each subject undergoes proper localization training 30 times in a day.
- Each subject must follow the same process till the experiment ends.
- The test is performed over several days to train the subject’s brain to locate the sound source accurately.

### 3.4. Data Collection

We collected sound localization tests every trial and day for multiple subjects in the Cartesian coordinate system. The data were automatically collected for target sound and estimated location of sound by subjects by using four Vicon cameras using the Nexus 1.8.3 program running on a personal computer. Once data were collected, these were analyzed by converting from the Cartesian to a Spherical coordinate system to better visualize distance, elevation, and azimuth. We analyzed distance error, elevation error, and azimuth error. The distance error refers to the distance between the target sound source location and the estimated location by subjects. The elevation error measures the ability to compute the angular displacement between the target sound source and the estimated location. The azimuth error is calculated based on the angular displacement of the target and estimated location. We calculated these three errors for each trial for each subject and on each day. We displayed these data graphically to improve the effectiveness of training progression and learning by each subject to improve the accuracy (reduces the error) in estimating the sound source location from trial to trial and day by day. We explored different types of feedback given to the subjects to observe the error reduction. We also tried to understand the ability of and variation in different subjects’ learning processes. This helped us refine our training over the months and find the best training concepts and steps for accurately estimating the sound source location.

### 3.5. Statistical Analysis

We investigated the training effectiveness by calculating each test location’s distance, elevation, and azimuth error. Equations (1)–(3) are the statistical representations to calculate the mean distance, elevation, and azimuth errors, respectively, for each day as training progresses. Table 1 defines all parameters involved in our research. The mean errors are graphically presented over the days in a line plot and box plots.

(1) $$D_{e}=\sum_{j=1}^{s}\frac{\sum_{i=1}^{n}\frac{\sqrt{{\left(x_{aij}-x_{oij}\right)}^{2}+{\left(y_{aij}-y_{oij}\right)}^{2}+{\left(z_{aij}-z_{oij}\right)}^{2}}}{n}}{s}$$

(2) $$\theta_{e}=\sum_{j=1}^{s}\frac{\sum_{i=1}^{n}\frac{\left|\theta_{aij}-\theta_{oij}\right|}{n}}{s}$$

(3) $$\emptyset_{e}=\sum_{j=1}^{s}\frac{\sum_{i=1}^{n}\frac{\left|\emptyset_{aij}-\emptyset_{oij}\right|}{n}}{s}$$

### 3.6. Pretest Result

We started our perceptual training with many different approaches. Some of these approaches are explained below:

1. Perceptual training with voice feedback provided directly by the experimenter: The experimenter guided the subject on how to reach the target by giving voice instructions such as “go left or right” and “move up or down”. We observed a very high error in all subjects’ distance, azimuth, and elevation. The distance error decreased to 798 mm, 788 mm, and 693 mm on Day 3. The elevation error observed on Day 1, Day 2, and Day 3 was 18°, 21°, and 18°, and the azimuth error was 29°, 35°, and 21°, respectively. This shows that training is ineffective for sound localization in blind subjects.
2. Perceptual training with kinesthetic assistance: We used an in-device vibrator that provided kinematic feedback to the subject based on their success or failure in locating the sound source. With this change, errors were significantly reduced as training progressed. Errors of 614 mm, 469 mm, and 458 mm were observed for distance, 15°, 13°, and 10° for elevation, and 28°, 26°, and 21° for azimuth for Day 1, Day 2, and Day 3, respectively.
3. Perceptual training with sound-guided feedback and active kinesthetic assistance: Figure 2 explains the added programmable speaker mechanism on device 1 in addition to the kinesthetic vibrator. With this enhancement, we observed that errors reduced drastically as trials and training progressed over the days due to continuous relative error feedback until the error reduced within 150 mm. From here onwards, detailed test results are explained that relate to our training method.

## 4. Result

**Figure 3:**
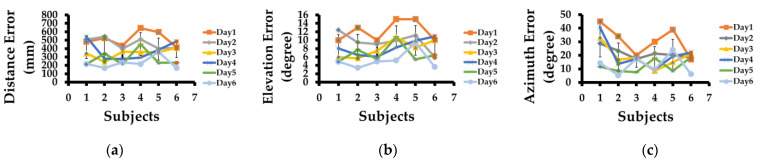
Subject’s performance in sound localization test. (**a**) Mean distance error, (**b**) mean elevation error, (**c**) mean azimuth error for each subject over six days of training.

In Figure 3, we present the result of the training of six subjects. All six subjects started with very poor estimation in locating a sound source. All subjects had different capabilities to locate a sound source. Hence, the initial distance errors were 482 mm, 524 mm, 436 mm, 645 mm, 597 mm, and 414 mm for subjects 1 to 6, respectively. After six days of sound localization training, these errors were reduced to 384 mm, 349 mm, 321 mm, 412 mm, 389 mm, and 352 mm, respectively as shown in Figure 3a. The elevation and azimuth errors also reduced for all six subjects as training progressed and are shown in Figure 3b,c. We note that subject 5 had the least improvement in estimating the sound source compared to other subjects, which is fine as behavioral learning is different for each human. However, the vital point from Figure 3a–c is that all subjects improved their ability to estimate the sound source location in terms of distance, elevation, and azimuth accurately by the end of training.

### 4.1. Distance Error

**Figure 4:**
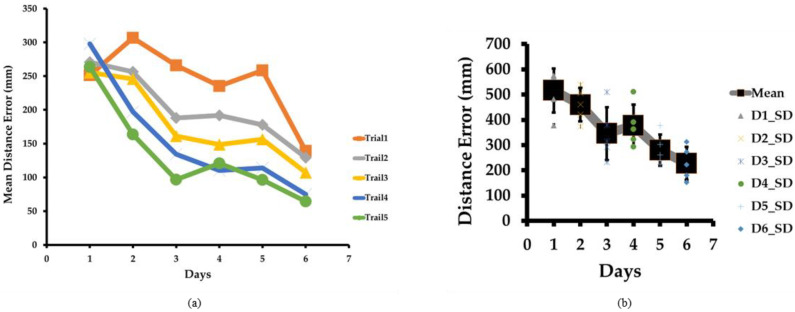
(**a**) Mean distance error in sound localization test as training progresses over the trials and days. Mean distance error reduces from trial 1 to 5 for each Day 1, 2, 3, 4, 5, and 6. (**b**) Box plot of mean distance error. The box plot shows that mean distance error and variation reduced gradually from Day 1 to Day 6, except for Day 5.

Figure 4a demonstrates subject performance regarding distance error in each trial and over the days. We can easily interpret from the line graph that as training progressed over the days, the distance error reduced, except for Day 1. On Day 1, the mean errors of all subjects for all test locations were 574 mm, 576 mm, 569 mm, 378 mm, and 483 mm for trials 1, 2, 3, 4, and 5, respectively. As subjects were very new to performing this task, the trial result was inconclusive on day 1, while on Day 2, the mean distance errors were 540 mm, 506 mm, 421 mm, 374 mm, and 461 mm for trials 1, 2, 3, 4, and 5, respectively. The mean distance error on Day 2 reduced as the trial progressed. The subjects were becoming trained as the trial progressed. Similar trends continued for Day 3, Day 4, Day 5, and Day 6. On Day 6, the mean distance errors were reduced to 312 mm, 273 mm, 222 mm, 181 mm, and 152 mm for trials 1, 2, 3, 4, and 5, respectively. Our internal goal was to reduce the mean distance error below 250 mm, which was achieved on Day 6. We stopped training on the sixth day. We can easily understand that the mean distance error reduced as the trial and days progressed. This proves our training method’s effectiveness in the estimation of the distance of sound sources by blindfolded subjects.

Figure 4b shows the overall mean distance error for six test locations for six subjects over five trials. The cumulative mean distance errors were 516 mm on Day 1, 460 mm on Day 2, 346 mm on Day 3, 377 mm on Day 4, 280 mm on Day 5, and 228 mm on Day 6. We stopped training when the errors reduced below 250 mm on Day 6 based on our internal set goal. The initial (first trial of Day 1) and final values (fifth trial of Day 6) of the mean distance errors were 574 mm and 152 mm, respectively. Our training method showed a 73% reduction in distance error, demonstrating the very high effectiveness of our training.

### 4.2. Elevation Error

**Figure 5:**
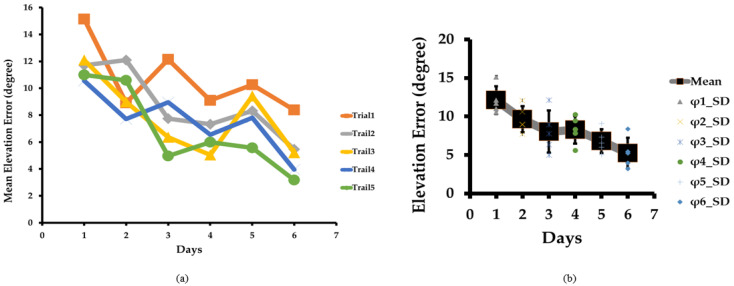
(**a**) Mean elevation error in sound localization test as training progresses over the trials and days. Mean elevation error reduces from trial 1 to 5 for each Day 1, 2, 3, 4, 5, and 6. (**b**) Box plot of mean elevation error. The box plot shows that mean elevation error and variation reduced gradually from Day 1 to Day 6.

Figure 5a illustrates our sound localization training and the mean elevation error for all test locations and subjects. For Day 1, the mean elevation errors were 15°, 12°, 12°, 11°, and 11° for trials 1, 2, 3, 4, and 5, respectively. These errors reduced to 8°, 5°, 5°, 4°, and 3° for trials 1, 2, 3, 4, and 5 respectively on Day 6. We can easily interpret that the mean elevation error gradually decreased as the trial and days progressed. It proves that our training method for the sound localization test is adequate for blindfolded subjects to estimate the sound source, as blind people are more sensitive to sound. It is expected that these results will be much better for blind people.

Similarly, the overall mean elevation error gradually reduced on each day from 12°, 10°, 8°, 8°, and 7°, to 5° for Days 1, 2, 3, 4, 5, and 6, respectively, with progress in training, as illustrated in Figure 5b. The initial and final values of the mean elevation errors were 15° and 3°, respectively. We achieved an 80% reduction in elevation error by the end of our training.

### 4.3. Azimuth Error

**Figure 6:**
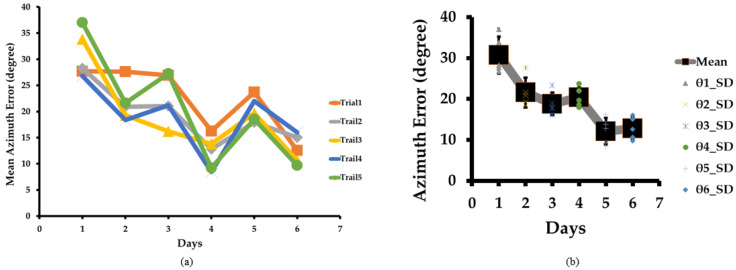
(**a**) Mean azimuth error in sound localization test as training progresses over the trials and days. Mean azimuth error gradually reduces from Day 1 to Day 6. (**b**) Box plot of mean azimuth error. The box plot shows that mean azimuth error and variation reduced gradually from Day 1 to Day 6, except for Day 5.

Figure 6a shows the sound localization tests and the mean azimuth error for all test locations for all subjects. On Day 1, the mean azimuth errors were very high at 28°, 28°, 34°, 27°, and 37° for trials 1, 2, 3, 4, and 5, respectively. On Day 1, as the trial progressed, the results were inconsistent, similarly to the mean distance error, as subjects began the training and were yet to locate the sound source accurately. However, by Day 6, the mean azimuth errors were reduced to 13°, 15°, 11°, 16°, and 10° for trials 1, 2, 3, 4, and 5, respectively. The mean azimuth error was reduced significantly, showing the training’s robustness similar to the mean distance and elevation errors.

The overall mean azimuth error was also reduced from 31°, 22°, 19°, 20°, and 12°, to 13° on each successive day, as shown in Figure 6b. The azimuth error slightly increased on Day 4 and Day 6 compared to Day 3 and Day 5, respectively. The rise in azimuth error was due to the fifth subject’s difficulty with concentrating. This is practically possible because sometimes the human brain behaves differently due to a lack of concentration. The initial and final values of the mean azimuth errors were 28° and 10°, respectively. Ultimately, we achieved a 64% reduction in azimuth error in six training days.

### 4.4. Bubble Plot

**Figure 7:**
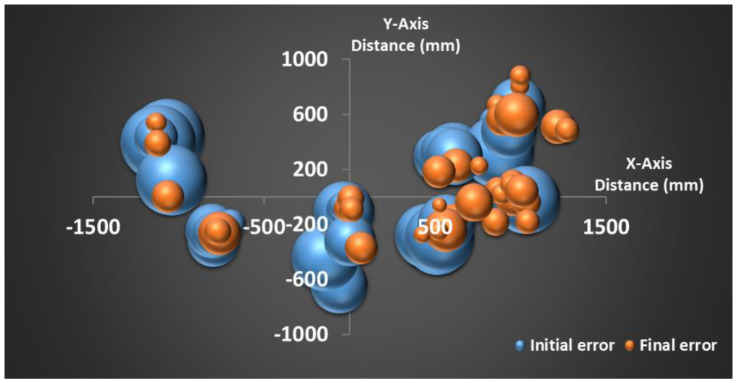
Bubble error with initial error (before training) in blue color and final error (after training) in orange color at various locations of the sound source in the X–Y plane.

We also developed bubble error plots for the initial (trial 1 of Day 1) and final (trial 5 of Day 6) errors, as shown in Figure 7, to the X–Y plane of the target sound source. In our experiment, the *z*-axis is always positive as the target sound is above the floor, so the bubble plot in the X–Y plane is good enough to show the relative initial and final errors. The location of the bubbles represents the sound source location in the X–Y plane and the size of the bubbles represents the relative errors. The bubble size (in orange color, after the training) of the final errors is much smaller compared to the bubble size (in blue color, before the training) of the initial errors in estimating the sound source location. The bubble plots also show that our test locations are scattered in the left, front, and right areas of the X–Y plane. 

### 4.5. Hypothesis Test

We tested each case’s hypothesis to develop a probability distribution curve. The null hypothesis was “error plot does not pass the normality test”, and the alternative hypothesis was “error plot passes the normality test”. We set the confidence level as 95%. With this confidence level, our hypothesis test is defined below.

*p*-value < 0.05: Null hypothesis is rejected and alternative hypothesis is accepted.

*p*-value > 0.05: Null hypothesis is accepted and alternative hypothesis is rejected.

**Figure 8:**
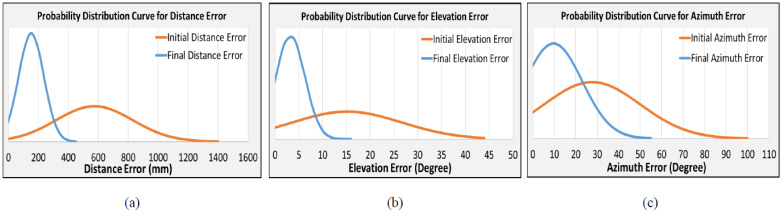
Probability distribution curve for (**a**) distance error, (**b**) elevation error, (**c**) azimuth error. The orange color represents the initial error prior to training and the blue color represents the final error at the end of training.

The orange color curve in Figure 8a shows the probability distribution curve of the initial (first trial of Day 1) distance error with a very high standard deviation of 256 mm. It has a *p*-value of 0.535 (>0.05), which means the null hypothesis is accepted and the alternative hypothesis rejected. The initial error on the first trial of Day 1 does not pass the normality test. After perceptual training skills with sound-guided feedback training over several days, the probability distribution curve (Figure 8a blue color curve) of the absolute distance error (fifth trial of Day 6) significantly reduces the standard deviation to a value of 84 mm. Hence, the probability distribution curve shrank with a lower variation with a mean error of 152 mm. The *p*-value was reduced to 0.004, <0.05. This indicates that the null hypothesis is rejected and the alternative hypothesis is accepted.

Similarly, we conducted a hypothesis test for the elevation and azimuth errors, as illustrated in Figure 8b,c. The standard deviation and *p*-value of the elevation error reduced from 12° and 0.05 to 3° and 0.00, respectively. Conversely, the standard deviation and *p*-value of the azimuth error reduced from 22° and 0.001 to 13° and 0.00, respectively. In both cases, the alternative hypotheses were accepted, and the probability distribution curve shrank with a lower variation and low mean error after the perceptual training with sound-guided feedback.

The proposed training was successfully applied to six blindfolded subjects with the internal goal of accurately locating the sound source in 3D space within 250 mm. We conducted the training for each subject for several days till the distance error fell below 250 mm. On each day, we performed the test for multiple trials to ensure subjects had enough activation of the brain to process and estimate the sound source. In each trial, we performed six different test locations to avoid any test method biasing. We found that all six subjects performed very well during the training. At the end of the sixth day, distance, elevation, and azimuth error were reduced by 73%, 80%, and 64%, respectively. The mean distance error was reduced from 574 mm to 152 mm. The mean elevation and azimuth errors were reduced from 15° to 3° and 28° to 10°, respectively.

To analyze our results further, we found that 60% of the time, subjects underestimated the distance to the sound source location, 54% of the time they estimated above the sound source, and 56% estimated in the right direction, as illustrated in Table 2. These results show that the subjects were very balanced in their estimation of the distance of the sound source and its location, considering up/down or left/right. To understand the result further, we divided the area in front (azimuth) of the subject into three parts. These sections were named left, center, and right. All test points lower than −15° in actual azimuth angle were named as left, in between −15° to 15° were named as the center, and those greater than 15° were named as the right side. We observed that more subjects (64%) underestimated the distance of the sound source on the left side. At the same time, subjects underestimated the distance up to 58% of the time in the center and 56% of the time on the right side. For elevation analysis, subjects located the sound source in the up direction for 68% of the time in the center. For left–right accuracy, subjects located the sound source fairly in each of the three areas.

We also divided all test points into near (for distances less than 1000 mm) and far (for distances more than 1000 mm) as summarized in Table 3. In the near area, 90% of the time, subjects underestimated the distance, and 68% of the time located the sound source location in the up direction. Past studies also found that the distance of near-distance subjects are underestimated more than far-distance subjects.

## 5. Discussion

To investigate the human ability to locate the 3D sound source and see the effect of guided training, we evaluated multi-modal training based on the sound-guided feedback of relative error with kinesthetic assistance. The proposed multi-modal training enabled subjects to locate the sound source through multi-modal sensory interaction with the environment and allowed subjects to practice through real-time feedback. Real-time feedback encouraged subjects to learn about their mistakes and allowed them to correct their errors. Our investigation revealed that real-time feedback on a relative error is essential for the effectiveness of training. In addition, as shown in Table 4, we found that kinematic assistance is essential in training performance. Apparently, the complexity of 3D sound localization, including azimuth, elevation, and distance, makes incorporating auditory feedback more desirable than kinesthetic feedback. We understand that spatial perception in our brain is hierarchical, where pathways from visual and auditory perception are integrated from the kinesthetic perception to form overall spatial perception in our cerebral cortex. Therefore, we conjecture that kinesthetic assistance effectively reinforces auditory feedback to strengthen spatial perception in our cerebral cortex [33,34]. In addition, sound-guided relative error feedback increases sensitivity to localization error, further strengthening spatial perception in our brains.

As shown in Table 4, we performed a comparative analysis of different types of feedback used in perceptual training for sound localization. It compared six different types of feedback used in sound localization training. Our initial method 1 (verbal feedback) and method 2 (kinesthetic feedback) showed very little improvement in mean distance, elevation, and azimuth accuracies. However, our proposed method 3 (sound-guided feedback with kinesthetic assistance) showed significant improvement compared to methods 1 and 2. Furthermore, we compared our proposed perceptual training method 3 with different types of training/feedback as used in related works [35,36]. We can infer from these results that kinesthetic with sound-guided feedback plays a crucial role in improving sound localization accuracy compared to other feedback and training methods. Table 4 illustrates that the proposed perceptual training method with kinesthetic and sound-guided feedback (method 3) significantly improves the accuracies in estimating the distance, elevation, and azimuth by 74%, 80%, and 64%, respectively, which are very high compared to other types of localization training.

**Table 4:** Sound localization comparative summary.

Sound Localization Training	Percentage Improvement in		
	Mean Distance Accuracy	Mean Elevation Accuracy	Mean Azimuth Accuracy
Method 1: Verbal feedback	13%	14%	28%
Method 2: Kinesthetic feedback only	25%	33%	25%
Method 3 (Proposed): Sound-guided feedback with kinesthetic assistance	74%	80%	64%
Trial and error without feedback [36]	44%	-	-
Visual and auditory feedback [35]	-	38%	
Visual, auditory, and haptic feedback [31]	-	-	17%

## 6. Conclusions and Future Works

This paper experimentally investigated how perceptual training can improve human 3D sound localization capability based on sound-guided feedback with kinesthetic assistance but without visual feedback. Unlike previous investigations, we considered the full 3D location, including distance and azimuth, and elevation angles, in our investigation. In particular, a novel perceptual training method that allows sound-guided relative error feedback was implemented to compare its effectiveness with conventional absolute error-based training methods. In addition, we discovered that human 3D sound localization capability could be improved by training, for which relative error feedback was proven to be more effective than absolute error feedback. We also discovered a tendency in biased 3D sound localization, depending on where the 3D sound source localization was performed, remaining intact after training. The incorporation of sound-based relative error feedback and kinesthetic assistance into training introduces more effective, guidance-based learning and promotes the development of well-honed human kinesthetic perception, leading to a better formation of neuroplasticity associated with 3D sound localization without visual aid. The subject of further research will be how training in 3D sound localization affects short-term and long-term memory in our brain based on neuroplasticity.

## Figures and Tables

**Table 1:** List of symbols with descriptions.

Symbol	Description
*x_a_*, *y_a_*, *z_a_*	Sound source location in Cartesian coordinates
*x_o_*, *y_o_*, *z_o_*	Estimated location by subjects in Cartesian coordinates
*d_a_*, *θ_a_*, *∅_a_*	Sound source location in spherical coordinates
*d_o_*, *θ_o_*, *∅_o_*	Estimated location by subjects in spherical coordinates
*d* *_aij_*	Actual distance of ith test for jth subject
*d* *_oij_*	Observed distance of ith test for jth subject
*D_e_*	Overall distance error in any trial on a day
*θ_aij_*	Actual elevation of ith test for jth subject
*θ* *_oij_*	Observed elevation of ith test for jth subject
*θ* *_e_*	Overall elevation error in any trial on a day
*∅* *_aij_*	Actual azimuth of ith test for jth subject
*∅* *_oij_*	Observed azimuth of ith test for jth subject
*∅_e_*	Overall azimuth error in any trial on a day
*i*	ith test point, which varies from 1, 2, ……, n
*j*	jth subject, which varies from 1, 2, ……, s
*n*	Total number of test points
*s*	Total number of subjects
*t*	Total number of trials
*u*	Total number of training days

**Table 2:** Left, center, and right analysis.

Parameters	All		Left (Azimuth < −15°)		Center (−15° ≤ Azimuth ≤ 15°)		Right (Azimuth > 15°)	
	Data Points	%	Data Points	%	Data Points	%	Data Points	%
Over distance Under distance Elevation—Up	427	40%	190	36%	70	42%	167	44%
	648	60%	336	64%	97	58%	215	56%
	576	54%	251	48%	114	68%	211	55%
Elevation—Down	499	46%	275	52%	53	32%	171	45%
Azimuth—Right	607	56%	294	56%	88	53%	224	59%
Azimuth—Left	468	44%	232	44%	79	47%	158	41%

**Table 3:** Near and far analysis.

Parameters	All		Near Distance (≤1000 mm)		Far Distance (>1000 mm)	
	Data Points	%	Data Points	%	Data Points	%
Over distance Under distance Elevation—Up	427	40%	16	10%	411	45%
	648	60%	139	90%	509	55%
	576	54%	105	68%	471	51%
Elevation—Down	499	46%	50	32%	449	49%
Azimuth—Right	606	56%	76	49%	530	58%
Azimuth—Left	469	44%	79	51%	390	42%

## Data Availability

Not applicable.
